# RETRACTED: Atta et al. Seawater Absorption and Adhesion Properties of Hydrophobic and Superhydrophobic Thermoset Epoxy Nanocomposite Coatings. *Nanomaterials* 2021, *11*, 272

**DOI:** 10.3390/nano16050300

**Published:** 2026-02-27

**Authors:** Ayman M. Atta, Mohamed H. El-Newehy, Meera Moydeen Abdulhameed, Mohamed H. Wahby, Ahmed I. Hashem

**Affiliations:** 1 Department of Chemistry, College of Science, King Saud University, Riyadh 11451, Saudi Arabia; melnewehy@ksu.edu.sa (M.H.E.-N.); meeranis@yahoo.co.in (M.M.A.); 2 Department of Chemistry, Faculty of Science, Tanta University, Tanta 31527, Egypt; 3 Chemistry Department, College of Science, Ain Shams University, Abasia, Cairo 11566, Egypt; chemist_wahby61@yahoo.com (M.H.W.); emyhashem2004@yahoo.com (A.I.H.)

The journal retracts the article titled “Seawater Absorption and Adhesion Properties of Hydrophobic and Superhydrophobic Thermoset Epoxy Nanocomposite Coatings” [1], cited above.

Following the publication, concerns were brought to the attention of the Editorial Office regarding the integrity and accuracy of the images and data presented in the published paper [1].

In accordance with the journal’s standard procedures, the Editorial Office and Editorial Board conducted an investigation that identified multiple indications of inappropriate image editing and panel duplication between this article and a significant number of previously published works by the same authorship group. These concerns relate to the images and data presented in Figures 4, 5, 7 and 8. Following further discussions with the authors, the Editorial Board was unable to verify the integrity of the figure in question based on the explanations and supplementary material provided. Consequently, the Editorial Board has lost confidence in the reliability of the overall findings and decided to retract this publication [1], as per MDPI’s retraction policy.

This retraction was approved by the Editor-in-Chief of the *Nanomaterials* journal. 

The authors have been informed of this retraction but did not provide a comment on this decision.

## References

[1] Atta A.M., El-Newehy M.H., Abdulhameed M.M., Wahby M.H., Hashem A.I. (2021). RETRACTED: Seawater Absorption and Adhesion Properties of Hydrophobic and Superhydrophobic Thermoset Epoxy Nanocomposite Coatings. Nanomaterials.

